# Population studies of sporadic cerebral amyloid angiopathy and dementia: a systematic review

**DOI:** 10.1186/1471-2377-9-3

**Published:** 2009-01-13

**Authors:** Hannah AD Keage, Roxanna O Carare, Robert P Friedland, Paul G Ince, Seth Love, James A Nicoll, Stephen B Wharton, Roy O Weller, Carol Brayne

**Affiliations:** 1 Department of Public Health and Primary Care, University of Cambridge, Cambridge, CB2 0SR, UK; 2 Division of Clinical Neurosciences, School of Medicine, University of Southampton, Southampton, SO16 6YD, UK; 3 School of Medicine, Case Western Reserve University, Cleveland, Ohio, 44106, USA; 4 Academic Unit of Pathology, University of Sheffield, Sheffield, S10 2RX, UK; 5 Bristol Neuroscience, University of Bristol, Bristol, BS8 1TD, UK

## Abstract

**Background:**

Deposition of amyloid-β (Aβ) in vessel walls of the brain as cerebral amyloid angiopathy (CAA) could be a major factor in the pathogenesis of dementia. Here we investigate the relationship between dementia and the prevalence of CAA in older populations. We searched the literature for prospective population-based epidemiological clinicopathological studies, free of the biases of other sampling techniques, which were used as a comparison.

**Methods:**

To identify population-based studies assessing CAA and dementia, a previous systematic review of population-based clinicopathological studies of ageing and dementia was employed. To identify selected-sample studies, PsychInfo (1806–April Week 3 2008), OVID MEDLINE (1950–April Week 2 2008) and Pubmed (searched 21 April 2008) databases were searched using the term "amyloid angiopathy". These databases were also employed to search for any population-based studies not included in the previous systematic review. Studies were included if they reported the prevalence of CAA relative to a dementia classification (clinical or neuropathological).

**Results:**

Four population-based studies were identified. They showed that on average 55–59% of those with dementia displayed CAA (of any severity) compared to 28–38% of the non-demented. 37–43% of the demented displayed severe CAA in contrast to 7–24% of the non-demented. There was no overlap in the range of these averages and they were less variable and lower than those reported in 38 selected sample studies (demented v non-demented: 32–100 v 0–77% regardless of severity; 0–50 v 0–11% for severe only).

**Conclusion:**

CAA prevalence in populations is consistently higher in the demented as compared to the non-demented. This supports a significant role for CAA in the pathogenesis of dementia.

## Background

Alzheimer's disease (AD) is the most common type of dementia and is characterised pathologically by the intraneuronal accumulation of neurofibrillary tangles (NFT) containing tau and ubiquitin, and by the extracellular accumulation of amyloid-β (Aβ) in brain tissue and in artery walls as cerebral amyloid angiopathy (CAA). Many studies have correlated the severity of dementia in AD with the number and distribution of NFTs, the number of plaques of insoluble Aβ, and the levels of soluble Aβ in the brain [as reviewed by [1]]. Relatively few studies, however, have investigated the relationship between dementia and the key pathological change of CAA.

CAA is the deposition of the amyloid peptides, of which Aβ is the most common, in the media and adventitia of small to medium-sized cerebral and leptomeningeal arteries, and less commonly in the walls of capillaries and veins [2-5]. The occipital lobe is most often involved, the frontal, parietal and temporal lobes less so and the cerebellum least; CAA is rare in the thalamus, basal ganglia and white matter [6,7]. Scholz [8] first emphasized the presence of CAA, suggesting that the pathological feature was associated with 'senility'. However, CAA was only reported to be a risk factor for dementia in the 1980s [9-11]. There has since then been increasing evidence to support this proposal [12,13] and CAA is now thought to play a significant role in the production of dementia [14-16]. Hereditary and sporadic types of CAA have been defined [17,18] however this review focuses on the common sporadic CAA found in the old.

The cause and effects of CAA have generated much debate especially in relation to dementia [19,20]. CAA represents the failure of elimination of Aβ along the perivascular pathways that serve as the lymphatic drainage channels for the brain [20]. Soluble tracers injected into the mouse striatum drain out of the brain along basement membranes of capillary and artery walls [21]. In CAA, Aβ is deposited in the very same perivascular drainage pathways outlined, in capillary and artery walls, in the injection studies in mice. This suggests that there is a failure of elimination of Aβ along ageing cerebral and leptomeningeal arteries [20,22,23]. Stiffening of artery walls with age and cerebrovascular disease may be a key element in reducing the elimination of Aβ from the brain in the elderly and in AD [20,24,25]. Transgenic mice that overproduce Aβ only in the brain develop CAA [19] which further supports the hypothesis that, in CAA, Aβ is entrapped in the perivascular pathways by which fluid and solutes drain from the brain [20,22].

Two major consequences arise from CAA. First: arteries weakened by deposits of Aβ in their walls tend to rupture and result in CAA-related intracerebral haemorrhage [26,27]. Second: blockage of perivascular drainage pathways by Aβ may be associated with accumulation of Aβ in the brain. Ultimately it is increased levels of soluble Aβ that correlates with cognitive decline in patients with AD [28,29]. It is possible that drainage of other soluble metabolites from the brain may also be impeded in CAA. This would result in a loss of homeostasis in the neuronal extracellular environment that could contribute to cognitive decline in AD [20].

CAA has been related to other neuropathological markers of dementia including neuritic plaques and NFTs [30-32]. The amount of phospho-tau in neurites was found to be greater in grey matter surrounding cerebral vessels affected by CAA than in grey matter away from affected vessels [33]. The relationship between CAA and other neuropathologies is not simple however, and there is great heterogeneity in CAA severity in brains with AD-type pathology [34]. CAA has been reported to affect cognition/dementia status independently of other neuropathological markers of dementia [3,7,12,35,36]. However, in one study CAA was found to be associated with dementia only in those who lacked any AD-type pathology [37].

Assessing the possible impact of CAA or any neuropathological marker of dementia is best done using a sample that closely reflects the population at risk [38]. The vast majority of studies assessing the relationship between CAA and dementia have assessed selected samples (e.g., necropsy and hospital patients) which do not reflect the population at risk, in terms of either clinical or age profiles. Many of these studies have had access to limited clinical information, which further limits the interpretation of results. Thus, although CAA has been reported to play a significant role in the causation of dementia, it is unknown whether this is partly an artefact of using highly selected samples and/or the predominant use of neuropathological dementia classifications such as AD (which most likely relates to CAA closer than clinical diagnoses). The role of CAA in dementia at a population level is uncertain.

Our aim in this study was to investigate the importance of CAA in relation to dementia in a population-based context. We have addressed this by systematically reviewing previous studies that have assessed the relationship between CAA and dementia in prospectively sampled population-based cohorts of elderly people. For comparison, we have systematically reviewed studies of CAA on selected samples.

## Methods

### Identifying population-based studies for review

A systematic review of population-based neuropathological studies of ageing and dementia in older people was published in 2006 [39]. Six studies were identified as being fully population-based – the Hisayama Study (Japan), Vantaa 85+ (Finland), the Cambridge City over 75 Cohort (CC75C; England), the Honolulu-Asia Aging Study (HAAS; USA), the Cache Country Study (USA) and the MRC Cognitive Function and Ageing Study (CFAS; England and Wales). To identify articles for the current review, PsychInfo (1806–April Week 3 2008), OVID MEDLINE (1950–April Week 2 2008) and Pubmed (searched 21 April 2008) databases were used to search for papers employing any of these six study populations to assess CAA. Study titles were searched for as keywords, along with 'amyloid angiopathy'. When no study could be found using the key term 'amyloid angiopathy', it was replaced by 'neuropathology' and the relevant articles were read and searched to determine if CAA was assessed. To ensure that no further prospective population-based clinicopathological studies had been published since Zaccai et al. [39], a search was conducted using the same databases detailed above using the terms 'population' and 'amyloid angiopathy' or 'pathology'. No new studies were identified.

### Identifying selected-sample studies for review

To compare CAA prevalence rates in population-based studies with those in studies of selected samples, we conducted a systematic review of studies in which CAA prevalence was determined relative to dementia status.

To identify studies using selected samples the PsychInfo (1806–April Week 3 2008), OVID MEDLINE (1950–April Week 2 2008) and Pubmed (searched 21 April 2008) databases were searched. One thousand three-hundred and seventy-three studies were identified using the search term 'amyloid angiopathy'. Titles and abstracts from these studies were read and studies were excluded if they did not assess human cases, the prevalence of CAA in the demented and/or non-demented specifically related to hereditary/familial CAA, reported a sample with a *n *less than 10, reported CAA prevalence relative to a condition other than dementia or only a feature of dementia (e.g., Parkinson's disease or *APOE *genotype), selected cases based on the presence of CAA, if they clearly reported the same cases as a previously selected study [e.g., [30,40,41]], if analyses were not neuropathological (e.g., MRI), or had been included as one of the population-based studies described above. The search was restricted to the English language. When a decision could not be made as to whether the article should be excluded, full articles were read. When articles were read, any cited articles that were not identified during the search were included.

## Results

### Summary of findings from population-based studies

Five of the six population-based studies reported CAA prevalence rates [13,14,35,42,43], of which four calculated these rates relative to dementia status (demented and/or non-demented) prior to death [all but [43]]. The findings from the four studies identified are reviewed below and summarised in Additional file 1. None of the studies assessed sex differences and only one study provided prevalence relative to age-groups [14] and thus prevalence relative to age and sex factors are not shown.

#### Cambridge City over 75 Cohort (CC75C)

Xuereb et al. [42] reported on CAA prevalence in the CC75C. The study comprised 99 individuals (68% women) over 80 years of age. CAA was assessed in meningeal and parenchymal areas of the occipital, frontal, temporal and parietal cortices as well as in the hippocampus. All CAA was recorded, regardless of severity. The authors reported that regardless of distribution, brains from participants with clinical dementia had a significantly higher prevalence of CAA (55%) than brains from participants who were not demented (26%).

#### MRC Cognitive Function and Ageing Study (MRC-CFAS)

MRC-CFAS [14] assessed the association between severe CAA and clinical dementia in 209 (57% female) individuals from England with a mean age of 86 years. Parenchymal and meningeal CAA was scored in the entorhinal, frontal, temporal, parietal and occipital cortices as well as the hippocampus. Severe CAA was present in 37% of the clinically demented and 7% of the non-demented, and was significantly associated with dementia (odds ratio/OR 9.3, 95%, confidence interval/CI 2.7–41.0) independent of other dementia-related neuropathologies.

#### Honolulu-Asia Aging Study (HAAS)

Pfeifer et al. [13] assessed CAA (controlling for other dementia-related neuropathologies) and clinical dementia in Japanese-American men in the HAAS. CAA was assessed in frontal, temporal, parietal and occipital cortices in 211 cases. Those with CAA were more likely to be older (86 versus 84 years of age) and carry at least one *APOE *ε4 allele (23% versus 6%) than were those without CAA. The prevalence of CAA regardless of severity did not vary significantly between the demented and non-demented, with rates of 55% and 38% respectively (significance value not provided). Accordingly, the authors concluded that CAA regardless of severity did not confer a significant risk for dementia. There was however a significant difference in the prevalence of severe CAA, with the demented having a higher prevalence (43%) than the non-demented (24%).

#### Vantaa 85+ study

Tanskanen et al. [35] assessed CAA in 74 (82% women) Finnish individuals over the age of 95 who were part of the Vantaa 85+ study. CAA severity was assessed in frontal, parietal, temporal, cingulate and cerebellar cortices, and an average severity calculated. As reported by Pfeifer et al. [13], those with CAA were more likely to have at least one *APOE *ε4 allele – 45% as compared to 8%. Of the clinically demented, 59% had CAA as compared to 28% of the non-demented. It was reported that moderate or severe CAA conferred a risk for dementia (OR not reported).

From the summaries and Additional file 1  it is evident that in population-based samples, the prevalence of CAA is higher in the demented than the non-demented. On average, 55–59% of those with clinical dementia had CAA (regardless of severity) compared to 28–38% of the non-demented. For severe CAA, prevalence appeared to decrease relatively equally across demented and non-demented groups, 37–43% of the clinically demented displaying severe CAA as compared to 7–24% of the non-demented. Only one study reported the prevalence of CAA to be non-significantly higher in demented than non-demented individuals however, this was for CAA regardless of severity. When only severe CAA was assessed, those with dementia had a higher prevalence than did the non-demented [13]. This suggests that in the population, severe CAA may be a better discriminator of clinical dementia than CAA regardless of severity.

### Summary of selected-sample studies

Thirty-eight studies were identified for review and are summarised in Additional file 2. It can be seen in Additional file 2 that the prevalence of CAA, regardless of severity, is higher in the demented (32–100% regardless of subtype or 47–100% for AD-only) than the non-demented (0–77%) in selected samples. The studies that assessed severe CAA reported prevalence rates of 0–50% in the demented (6–50% for AD-only) and 0–11% in the non-demented. Only five of the thirty-eight selected studies tested for an association between CAA and dementia [34,37,40,44,45]. All of those that did reported that cases with dementia had a significantly higher prevalence than those without, or that CAA prevalence correlated significantly with dementia. The only exception was the study by Jellinger and Attems [40] in which this was true for capillary CAA but not other forms of CAA.

## Discussion

### How do CAA prevalences differ between population-based and selected-sample studies?

There was greater variability in CAA prevalence rates in selected-sample studies than population-based studies. This may be due to differential bias between selected samples and the paucity of clinical information prior to death in most studies.

Estimates of CAA prevalence in the population studies were lower than in those from selected samples. This may be due to diagnostic differences (primarily neuropathological methods in studies of selected samples as compared to clinical methods in population-based studies) and younger populations being assessed, perhaps including some cases of hereditary rather sporadic CAA. Only eight studies of selected samples employed a clinical diagnosis of dementia rather than a neuropathological confirmation of AD or VaD. This is an important difference from population-based studies, all of which employed a clinical diagnosis, as neuropathological and clinical AD classification methods correspond imprecisely [46,47].

The mean age of the cohorts in selected-sample studies was between 69–91, with participants in their 40s and 50s commonly included [e.g., [44,48-50]]. These studies on selected samples therefore included much younger cases than population-based studies. Given that population-based studies most closely reflect the population at risk of disease, these findings highlight their important contribution to the understanding of pathological correlates of clinically determined dementia relevant to populations and patients. Evidence relating to the pathology of dementia needs to be obtained from both population-based as well as from selected samples.

Both the population-based studies and those on selected samples employed various stains to assess CAA, including Congo red, thioflavin-S, anti-Aβ immunohistochemistry and Weigert's haematoxylin. Some studies used multiple stains – either different stains for different cases [e.g., [51]] or anti-Aβ immunostaining to validate Congo red-positive cases [e.g., [34]]. To the authors' knowledge, no study has directly compared the sensitivity and specificity of these staining methods in relation to the measurement of CAA. Haglund and Englund [49] assessed a small sample (*n *= 10) with both Congo red and anti-Aβ immunohistochemistry. They reported that for 8 of the 10 cases, CAA severity appeared similar with both methods (Aβ immunohistochemistry perhaps giving slightly higher severity grades), while the other 2 cases showed little Congo red positivity but strong labelling of vascular Aβ.

It is not only the staining method that may produce variability between studies, the pre-treatment methods and sampling strategies also contribute [52,53]. The selection of parenchymal and/or leptomeningeal samples is also important, as CAA severity is usually higher in the later [54]. The choice of cortical region(s) and the grading system employed in the scoring of CAA in terms of its scale (e.g., 0–3 or 0–4) and definition of severity, would have also contributed to inter-study variability.

### CAA at the population level including risk factors

At the population level, it appears as though CAA prevalence rates are consistently higher in the demented than the non-demented, suggesting that CAA is an important dementia-related abnormality in a population context, particularly severe CAA. This finding is pertinent in light of recent reports of overlap in the prevalence rates between demented and non-demented cohorts of other dementia-related abnormalities such as plaques and tangles [14,47].

Determination of the presence and severity of CAA may aid in neuropathological assessment that more closely resembles the clinical assessment of dementia. The MRC-CFAS study reported the OR for risk of a clinical dementia diagnosis to be higher for severe CAA (OR = 9.3, 95%CI 2.7–41.0) than either neocortical neuritic plaques (OR = 5.0, 95%CI 1.2–29.8) or neocortical tangles (OR = 4.6, 95%CI 1.5–15.8) [14]. Future work should elucidate whether the inclusion of CAA in the neuropathological assessments of dementias such as AD improves the specificity and sensitivity of classification relative to clinical dementia during life. Further, given that CAA is a potential discriminator of dementia from normal ageing, imaging methods that detect CAA in living individuals may aid in the differential diagnosis of clinical dementia syndromes [5,12,55]. CAA is a potential predictor of the development of dementia during life, for which treatment and management strategies might be developed. The presence of CAA in the living non-demented may also indicate a specific syndrome such as Vascular Cognitive Impairment No-Dementia (VCI-ND) [56] – this also needs to be investigated in the future.

Some studies [6,34,43,57-60] but not all [14,48,61] have found that increasing age in the elderly is a risk factor for CAA. In a community sample of 100 individuals 50–91 years, Mastaglia et al. [62] found CAA prevalence to increase with age to a maximum in those over 90. As yet there is no evidence that the prevalence of CAA varies with sex [7,43]. Cerebrovascular disease (e.g., atherosclerosis) is another possible risk factor for CAA. It has been suggested to affect the efficiency of Aβ removal by perivascular drainage, leading to CAA [63]. However, hypertension, diabetes mellitus and hyperlipidemia do not appear to be risk factors for CAA [60].

The ε4 allele of the *APOE *gene has been reported to be a risk factor for CAA as it is for AD [64-68] and this was seen in two of the population-based studies reviewed here [13,35]. It has been suggested that the association between the *APOE *ε4 allele and CAA is due to the ε4 allele being related to vascular rather than parenchymal accumulation of Aβ [64]. The APOE ε4/ε4 genotype has been associated with CAA-related inflammation [69], while the ε2 allele has been reported to be a risk factor for CAA-related haemorrhage in both those with and those without AD [65,70].

### How clinical studies can inform these findings

Studies of clinic and/or necropsy cohorts are valuable for determining the implications of CAA at a population level. For instance, these studies have found that many neurological events induced by CAA can themselves compromise neurological integrity and cognitive function. For example, CAA increases vessel wall fragility and increases the risk of cortical and subcortical intracerebral haemorrhage [5,71], carrying a high risk of poor neurological and cognitive outcome [72]. CAA may impair blood flow, leading to ischemic damage to the cerebral cortex and white matter, which is also associated with neurological and cognitive impairment [73-75]. CAA is associated with failure of drainage of interstitial fluid from cerebral white matter in AD and the pathogenesis of leukoaraiosis [76]. Further, the Aβ peptide that accumulates in CAA can provoke inflammation/vasculitis in a small subset of individuals [69,77] with both microglia and T-cells involved [78]. CAA-related inflammation has also been proposed to be related to severe cognitive decline due to circulatory dysfunction [69,78]. Thus, patients who develop haemorrhage, ischaemia, white matter damage or inflammation as complications of CAA are at risk of further cognitive impairment.

The type of vessel involved and distribution of CAA may differentially affect cognition. It was reported that capillary CAA is more closely associated with impaired cognition than is CAA of larger blood vessels [30,79]. Further, although CAA is most prevalent in the posterior regions of the cerebral hemispheres, the association between CAA and clinical dementia has been reported to be strongest for frontal CAA [37].

### Possible limitations and future directions

The four population-based studies reviewed here employed relatively crude measures of CAA. There were varying attempts to take into account the interactions with a wide range of other dementia-related pathologies and the severity of CAA. No study took into account the distribution of CAA, the presence of inflammation and the type of vessel affected. All these factors have been reported to influence the association with cognition [e.g., [30,37,79,80]].

Population-based studies, as with studies on selected samples, face some important stumbling blocks in the investigation of CAA. The distribution of CAA is variable [4] and its detection critically dependent of the extent and distribution of histological sampling. There is likely to be an under-diagnosis of CAA even in severe cases [18]. There is currently no consensus as to how to sample for or detect CAA or grade its severity. Full evaluation of CAA is most satisfactorily assessed by isolating cerebral and leptomeningeal vessels from the brain and staining for amyloid with Thioflavin [22,76]. A standard consensus method for detecting and classifying CAA would greatly facilitate future population-based multi-centre studies of CAA and such plans are underway.

## Conclusion

CAA prevalence rates are higher in demented than non-demented old people in prospective population-based studies. This suggests that CAA is a more significant dementia-related abnormality than previously recognised and underscores the need to understand the aetiology and pathogenesis of CAA and its contribution to dementia in the population.

## Competing interests

The authors declare that they have no competing interests.

## Authors' contributions

All authors drafted and edited the manuscript, HADK also carried out the systematic review.

## Pre-publication history

The pre-publication history for this paper can be accessed here:



## Supplementary Material

Additional file 1 **Additional table 1.** Prevalence (to nearest whole number) of CAA in population-based studies regardless of severity and relative to severe CAA only in the demented and non-demented, as well as the significance of association between CAA and clinical dementia. Click here for file

Additional file 2 **Additional table 2.** Prevalence (to nearest whole number) of CAA in studies using selected non-population based samples in the demented and non-demented, as well as the significance of association between CAA and clinical dementia (if given) ordered by date of publication. Click here for file
