# Supplementary material for: Population studies of sporadic cerebral amyloid angiopathy and dementia: a systematic review
Source: BMC Neurol. 2009 Jan 13;9:3. doi: 10.1186/1471-2377-9-3 (PMC2647900; doi:10.1186/1471-2377-9-3)
Supplement: Additional file 1 — Additional table 1. Prevalence (to nearest whole number) of CAA in population-based studies regardless of severity and relative to severe CAA only in the demented and non-demented, as well as the significance of association between CAA and clinical dementia. [file 1471-2377-9-3-S1.doc]

**Additional table** **1** - Prevalence (to nearest whole number) of CAA in population-based studies regardless of severity and relative to severe CAA only in the demented and non-demented, as well as the significance of association between CAA and clinical dementia.

| Study | Population sample | Mean age at death (SD if available) | Age range at death | % of women | Dementia diagnosis | Stain | Region(s) | Number of cases | | Prevalence of CAA (%) in sample | | Test for association between CAA and  dementia | |
| --- | --- | --- | --- | --- | --- | --- | --- | --- | --- | --- | --- | --- | --- |
|  |  |  |  |  |  |  |  | Demented | Non-demented | Demented | Non-demented | OR | χ^2^ |
| Regardless of severity | | | | | | | | | | | | | |
| Xuereb et al. (2000) | Cambridge, UK | Unknown | Unknown, but all 80+ | 68 | Clinical | Congo-red | Frontal, temporal, parietal, occipital and hippocampus^ | 47 | 52 | 55 | 26 |  | *p*=.003 |
| Pfeifer et al. (2002)* | Honolulu, USA | 85 (5) | Unknown | Unknown | Clinical | Anti-Aβ | Frontal, temporal, parietal and occipital | 78 | 138 | 55 | 38 |  | Non-sig. |
| Tanskanen et al. (2005) | Vantaa, Finland | 97 | 95-107 | 82 | Clinical | Congo-red | Frontal, temporal, parietal, cerebellum and hippocampus | 49 | 25 | 59 | 28 |  | *p*=.026** |
| Severe only | | | | | | | | | | | | | |
| MRC CFAS (2001) | Multicentre, England and Wales | 86 (median not mean) | 70-103 | 57 | Clinical | Congo-red | Frontal, temporal, parietal, occipital and hippocampus and entorhinal^^ | 100 | 109 | 37 | 7 | 9.3  (95% CI 2.7-41.0)  *p* <0·001 |  |
| Pfeifer et al. (2002)* | Honolulu, USA | 85 (5) | Unknown | Unknown | Clinical | Anti-Aβ | Frontal, temporal, parietal and occipital | 73 | 138 | 43 | 24 |  | Sig., but *p* not given |

CI = confidence interval

*Note, appears twice

**Only moderate and severe CAA was included in this analysis, however, prevalence rates are regardless of severity

^Information from study webpage www.cc75c.group.cam.ac.uk

^^Information from study webpage www.cfas.ac.uk
