# Supplementary material for: Population studies of sporadic cerebral amyloid angiopathy and dementia: a systematic review
Source: BMC Neurol. 2009 Jan 13;9:3. doi: 10.1186/1471-2377-9-3 (PMC2647900; doi:10.1186/1471-2377-9-3)
Supplement: Additional file 2 — Additional table 2. Prevalence (to nearest whole number) of CAA in studies using selected non-population based samples in the demented and non-demented, as well as the significance of association between CAA and clinical dementia (if given) ordered by date of publication. [file 1471-2377-9-3-S2.doc]

**Additional table 2** - Prevalence (to nearest whole number) of CAA in studies using selected non-population based samples in the demented and non-demented, as well as the significance of association between CAA and clinical dementia (if given) ordered by date of publication.

| Study | Sample | Mean age at death (SD if available) | Age range at death | % of women | Dementia diagnosis | Stain | Region(s) | Number of cases | | Prevalence of CAA (%) in sample | | Test for association between CAA and dementia |
| --- | --- | --- | --- | --- | --- | --- | --- | --- | --- | --- | --- | --- |
|  |  |  |  |  |  |  |  | Demented | Non-demented | Demented | Non-demented |  |
| Regardless of severity | | | | | | | | | | | | |
| Mandybur [31] + | Clinical | 69 | 55-86 | 60 | Clinical | Congo red | Whole brain sections | 15 | 0 | 87 | n/a |  |
| Jellinger [81] | Clinical | Unknown | Unknown | Unknown | Neuropath | Congo red and Bodian silver | Unknown | 92  (AD only) | 0 | 78 | n/a |  |
| Glenner et al. [82] | Clinical | 69 | 43-89 | Unknown but both | Clinical confirmed by neuropath | Varied including congo-red, haematoxylin and eosin and Bielschowsky’s silver | Varied across cases but hippocampus whenever possible | 45  (AD only) | 16 (hyper-tensive) | 89 | 0 |  |
| Mountjoy et al. [61] | Clinical | 76 | 57-93 | 60 | Clinical | Congo red | Frontal, temporal, parietal and occipital | 15  (AD only) | 30 | 60 | 40 |  |
| Esiri and Wilcock [48]+ | Clinical | 80-82 (varied between groups) | 51-102 | 53 | Clinical | Congo red | Varied including frontal, temporal, parietal and occipital and hippocampus | 86  (45 AD  41 non-AD) | 73 | 58  (82 AD  32 non-AD) | 33 |  |
| Bergeron et al. [44]+ | Brain bank | 73 (8) case/ 71 (10) control | 43-92 | Unknown | Mixed | Congo red | Frontal, temporal, parieto-occipital and cerebellum | 30  (AD only) | 30 | 86 | 40 | χ^2^  *p*=.0009 |
| Yamanda et al. [60] | Brain bank | 83 | 59-101 | 63 | Neuropath | Congo red  (and Anti-Aβ for 4 cases) | Frontal, parietal, temporal, occipital, central grey matter, cerebellum | 43  (17 AD  26 non-AD) | 80 | 60  (88 AD  42 non-AD) | 55 |  |
| Joachim et al. [83] | Community | Approx 77 | Approx  53-97 | Approx 46 | Clinical confirmed by neuropath | Congo red | Occipital and one other | 131  (AD only) | 0 | 100 | n/a |  |
| Dickson et al. [84] | Community | Approx 90 | Unknown | Unknown | Clinical | Anti-Aβ | Occipital and frontal | 0 | 14 | n/a | 36 |  |
| Lopez et al. [85] | Clinical | 72 (8) | 56-86 | 48 | Clinical confirmed by neuropath | Congo red | Frontal, temporal, parietal and occipital and cerebellum | 40  (AD only) | 0 | 55 | n/a |  |
| Wu et al. [86] + | Clinical | 73-81 (varied between groups) | Unknown | 50 | Neuropath | Thioflavin-S | Five cortical regions (unknown) | 101  (33 LBD,  34  LBD+AD, 34 AD) | 34 | Parenchymal  75  (43 LBD, 85 LBD+AD, 97 AD)  Leptomening  81  (58 LBD, 85 LBD+AD, 100 AD) | Parenchymal  38  Leptomening  50 |  |
| Ellis et al. [51] | Clinical | Approx 78 | Unknown | 36 | Clinical confirmed by neuropath | Varied between Congo red, thioflavin-S and anti-Aβ | Hippocampus, entorhinal, frontal, temporal and parietal | 117 | 0 | 83 | n/a |  |
| Ogeng’o et al. [87]+ | Clinical | Unknown | 45-84 | Unknown | Neuropath | Anti-Aβ | Frontal, temporal, parietal, occipital, putamen and hippocampus | 0 | 52 | n/a | 17 |  |
| Olichney et al. [88] | Brain bank | 65-82 (varied between groups) | Unknown | 50 | Clinical confirmed by neuropath | Thioflavin-S | Frontal, temporal, parietal and hippocampus | 126  (84 AD,  42 LBD) | 0 | 70  (79 AD,  52 LBD) | n/a |  |
| Premkumar et al. [67] | Brain bank | 70-79 (varied between groups) | 55-90 | 55 | Neuropath | Congo-red and Anti-Aβ | Frontal, temporal, parietal and occiptal | 190  (AD only) | 16 | 96 | 0 |  |
| Davis et al. [89] | Community | 84 (7) | 69-100 | 54 | Clinical | Haematoxylin and eosin as well as anti-Aβ and Bielschowsky’s silver | Frontal, temporal, parietal, occipital and hippocampus | 0 | 59 | n/a | 77 |  |
| Zarow et al. [90]+ | Brain bank | 77 (9) | 57-93 | 50 | Neuropath | Thioflavin-S | Frontal, parietal, temporal and hippocampus | 101  (AD only) | 0 | 81 | n/a |  |
| Del Ser et al. [91] | Community | 77 | Unknown | 50 | Neuropath | Congo red | Frontal, parietal, occipital, temporal, hippocampus, insula, parahippocampus, basal ganglia, midbrain | 64  (35 AD,  11 LBD,  18 AD+LBD) | 0 | 42  (47 AD, 10 LBD, 50 AD+LBD) | n/a |  |
| Haglund & Englund [49] | Clinical | 76-85 (varied between groups) | 53-98 | 56 case/ unknown control | Clinical confirmed by neuropath | Congo red (and anti-Aβ for 10 cases) | Frontal | 63  (37 AD,  15 AD+VaD,  11 VaD) | 10 | 68  (70 AD,  67 AD+VaD,  64 VaD) | 20 |  |
| Yamada et al. [34] | Clinical | 86 (8) case/86 (8) control | 62-104 | 60 | Neuropath | Congo red and anti-Aβ (for confirmation) | Unclear, but most likely hippocampus and superior temporal gyrus | 82  (AD only) | 119 | 87 | 35 | χ^2^  *p*<.0001 |
| Chalmers et al. [64] + | Brain bank | 79 (9) case/75 (8) control | 58-97 | 53 | Neuropath | Anti-Aβ | Frontal, temporal and parietal | 125  (AD only) | 53 | 95 | 36 |  |
| Jellinger & Attems [92] + | Clinical | 83 (10) case/84 (8) control | 58-99 | 59 | Clinical confirmed by neuropath | Anti-Aβ | Neocortex, limbic, brainstem and cerebellum | 173  (AD only) | 130 | 97 | Unknown |  |
| Jellinger & Mitter-Ferstl [93] | Clinical | 82 (4) case/ 83 (9) control | 57-103 | 62 | Clinical confirmed by neuropath | Anti-Aβ | Neocortex, limbic, basal ganglia, brainstem and cerebellum | 730  (AD only) | 535 | 98 | Unknown |  |
| Love et al. [58] | Brain bank | Unknown | 60-102 | 49 | Neuropath | Anti-Aβ | Temporal and frontal | 0 | 152 | n/a | 32 |  |
| Mastaglia et al. [62] | Coroner | 75.1 | 50-91 | 40 | Clinical | Anti-Aβ | Frontal, temporal, parietal, occipital and hippocampus | 0 | 100 | n/a | Leptomening: 25  cortical: 18 |  |
| Tian et al. [94] | Brain bank | 74 (10) | Unknown | 48 | Clinical confirmed by neuropath | Anti-Aβ | Frontal, temporal, occipital and parietal | 69  (AD only) | 0 | 100 | n/a |  |
| Zekry et al. [3] | Clinical | 87 (6) | 79-101 | 79 | Clinical | Hematozylin and eosin, congo red, bodian silver, luzol fast blue and Anti-Aβ | Hippocampus, parahippocamus, temporal, frontal and substantia niagra | 24 | <10 | 100 | n/a |  |
| Haglund et al. [95] | Clinical | 84 (9) VaD/83 (4) AD | Unknown | 55 | Clinical confirmed by neuropath | Anti-Aβ | Temporal | 22  (11 VaD,  11 AD) | 0 | 82  (100 AD,  64 VaD) | n/a |  |
| Tian et al. [96] | Brain bank | 73-74 (varied between groups) | Unknown | 53 | Clinical confirmed by neuropath | Weigert’s haematoxylin and eosin | Frontal, temporal, occipital and parietal | 94  (AD only) | 0 | 100 | n/a |  |
| Tian et al. [71]+ | Brain bank | 73 (9) | Unknown | 54 | Clinical confirmed by neuropath | Weigert’s haematoxylin and eosin | Frontal, temporal, occipital and parietal | 137 (AD only) | 0 | 100 | n/a |  |
| Haglund et al. [97] | Clinical | 82 (9) | Unknown | 50 | Neuropath | Hematozylin and eosin, anti-Aβ  and congo red | Parietal and temporal | 26  (VaD only) | 0 | General: 73  (Capilllary: 8) | n/a |  |
| Jellinger & Attems [40]** | Brain bank | 84 (9) | 60-100 | Unknownbut both sexes | Neuropath | Anti-Aβ | Frontal | 100 in total (demented and non-demented) | | 88 | 56 | Correlation  with diagnosis: General: non-sig  Capillary: *p*<.01 |
| Tian et al. [98]+ | Brain bank | 75 | Unknown | 51 | Clinical confirmed by neuropath | Anti-Aβ | Frontal and occipital | 70 (AD only) | 0 | Occipital: 97  Frontal: 92 | n/a |  |
| Attems et al. [37] | Clinical | 84 (9) | 54-104 | 60 | Clinical | Anti-Aβ | Frontal, frontobasal, hippocampal and occipital regions | 96 | 75 | 77 | 57 | χ^2^  *p*<.006 |
| Jellinger et al. [99] | Clinical | 78 (7) | 60-103 | 66 | Clinical confirmed by neuropath | Anti-Aβ | Frontal, occipital, temporal, limbic, basal ganglia, brain stem, cerebellum and hippocampus | 371  (AD only) | n/a*** | 96 | n/a |  |
| Sonnen et al. [45] | Community | Unknown | 65+ | 57 | Clinical | Congo red^#^ | Occipital^#^ | 75 | 136 | 38 | 13 | Regression  *p*=.015 |
| Attems et al. [100] | Brain bank | Approx 79 | Approx  60-108 | Approx 60 | Neuropath | Anti-Aβ | Frontal, hippocampal and occipital | 760  (AD only) | 0*** | 98 | n/a |  |
| Jellinger & Attems [101] | Brain bank | 80 (5) | 65-96 | 50 | Clinical confirmed by neuropath | Anti-Aβ | Cortex, basal ganglia, brainstem and cerebellum | 20  (LBD only) | 0 | General: 65  Capillary: 90 | n/a |  |
| Severe only | | | | | | | | | | | | |
| Mandybur [31] + | Clinical | 69 | 55-86 | 60 | Clinical | Congo red | Whole brain sections | 15 | 0 | 13 | n/a |  |
| Esiri and Wilcock [48]+ | Clinical | 80-82 (varied between groups) | 51-102 | 53 | Clinical | Congo red | Varied including frontal, temporal, parietal and occipital and hippocampus | 86  (45 AD  41 non-AD) | 73 | 16  (25 AD  7 non-AD) | 3 |  |
| Bergeron et al. [44]+ | Brain bank | 73 (8) case/ 71 (10) control | 43-92 | Unknown | Mixed | Congo red | Frontal, temporal, parieto-occipital and cerebellum | 30  (AD only) | 30 | 50 | 10 | χ^2^  *p*<.002 |
| Wu et al. [86] + | Clinical | 73-81 (varied between groups) | Unknown | 50 | Neuropath | Thioflavin-S | Five cortical regions (unknown) | 101  (33 LBD,  34  LBD+AD, 34 AD) | 34 | Parenchymal  6  (0 LBD,  4 LBD+AD, 6 AD)  Leptomening  22  (3 LBD, 33 LBD+AD, 30 AD) | Parenchymal  0  Leptomening  3 |  |
| Olichney et al. [102] | Clinical | 80 | Unknown | 47 | Clinical confirmed by neuropath | Thioflavin-S | Frontal, temporal, parietal and hippocampus | 145  (AD with hypertension only) | 0 | 19 | n/a |  |
| Ogeng’o et al. [87]+ | Clinical | Unknown | 45-84 | Unknown | Neuropath | Anti-Aβ | Frontal, temporal, parietal, occipital, putamen and hippocampus | 0 | 52 | n/a | 2 |  |
| Zarow et al. [90] | Brain bank | 77 (9) | 57-93 | 50 | Neuropath | Thioflavin-S | Frontal, parietal, temporal and hippocampus | 101  (AD only) | 0 | 29 | n/a |  |
| Jellinger & Attems [92] + | Clinical | 83 (10) case/84 (8) control | 58-99 | 59 | Clinical confirmed by neuropath | Anti-Aβ | Neocortex, limbic, brainstem and cerebellum | 173  (AD only) | 130 | 26 | Unknown |  |
| Tian et al. [71] | Brain bank | 73 (9) | Unknown | 54 | Clinical confirmed by neuropath | Weigert’s haematoxylin and eosin | Frontal, temporal, occipital and parietal | 137 (AD only) | 0 | 36 | n/a |  |
| Tian et al. [98] | Brain bank | 75 (9) | Unknown | 51 | Clinical confirmed by neuropath | Anti-Aβ | Frontal and occipital | 70 (AD only) | 0 | Occipital: 46  Frontal: 18 | n/a |  |
| Chalmers et al. [64]+ | Brain bank | 79 (9) case/75 (8) control | 58-97 | 53 | Neuropath | Anti-Aβ | Frontal, temporal and parietal | 125  (AD only) | 53 | 14 | 11 |  |
| Jellinger & Attems [92]+ | Clinical | 83 (10) case/84 (8) control | 58-99 | 59 | Clinical confirmed by neuropath | Anti-Aβ | Neocortex, limbic, brainstem and cerebellum | 173  (AD only) | 130 | 26 | 0 |  |
| Jellinger & Mitter-Ferstl [93]+ | Clinical | 82 (4) case/ 83 (9) control | 57-103 | 62 | Clinical confirmed by neuropath | Anti-Aβ | Neocortex, limbic, basal ganglia, brainstem and cerebellum | 730  (AD only) | 535 | 24 | 0 |  |

CI = confidence interval

n/a = not applicable

LBD = Lewy Body Dementia

VaD = Vascular Dementia

+ Appears twice

^^ Information obtained from a personal communication with Dr Joshua Sonnen (9 May 2008)

* Results also presented in [103]

** Results also presented in [30, 40, 41]

***There was a larger sample that included non-demented participants, but the exact number and prevalence of CAA relative to this group was not given
